# Comparison of HemoFoam® and Conventional Gauze Dressing on Hemostasis of Vascular Access Site in Hemodialysis Patients

**DOI:** 10.31661/gmj.v8i0.1395

**Published:** 2019-07-23

**Authors:** Davood Bizari, Hadi Khoshmohabat, Soheila Salahshour Kordestani, Rouhollah Zarepur

**Affiliations:** ^1^Trauma Research Center, Baqiyatallah University of Medical Sciences, Tehran, Iran; ^2^Biomaterial Group, Medical Engineering Department, Amir Kabir University of Technology, Tehran, Iran

**Keywords:** Hemodialysis Patients, Hemostasis, Hemostatics, Bandages, Arteriovenous Fistula

## Abstract

**Background:**

Dialysis access puncture wound bleeding after needle extraction at the end of each hemodialysis session is a very important problem. This study evaluated the effect of HemoFoam^®^ compared to conventional gauze dressing on hemostasis of dialysis access puncture wound bleeding in hemodialysis patients.

**Materials and Methods:**

This one-group, before-after, clinical-trial was conducted on 60 hemodialysis patients selected by convenience sampling who underwent hemodialysis through arteriovenous fistula in Shahid Rahnemoon Hospital, Yazd, Iran in 2017. After reviewing the eligibility criteria, the study was performed in two separate sessions. In the first session, only HemoFoam^®^ was used while in the second session; the only conventional dressing was used. Time of hemostasis in each puncture wound was evaluated. Data were analyzed by SPSS 22 (IBM SPSS Statistics for Windows, Armonk, NY: IBM Corp, United States) using paired T-test and Chi-square tests.

**Results:**

The mean age of the patients was 55.20±14.25 years. Hemostasis was achieved in 76.6% of cases at the arterial access site in the first two minutes in the HemoFoam^®^ group. The mean homeostasis time in the HemoFoam^®^ group was 2.86±1.87 min at the venous access site and 3.15±1.97 min at the arterial access site (P<0.001). The mean homeostasis time in the conventional dressing group was 10.54±6.65 min at venous access site and 12.74±9.28 min at the arterial access site, which was significantly different between the two groups (P<0.001).

**Conclusion:**

HemoFoam^®^ is effective in reducing the time of homeostasis in the vascular access site of hemodialysis patients. Therefore, its use in hemodialysis wards is recommended for hemostasis in the dialysis access puncture wound bleeding.

## Introduction

Hemostasis is a complex process that can reduce the bleeding time in vascular damages. Different materials and events are involved in this process. Indeed, four events are involved in the process of hemostasis: vascular contraction, platelet plaque formation, fibrin formation, and fibrinolysis [1]. There are several ways to control bleeding such as direct pressure with conventional dressing or using a tourniquet. Often, these methods are not sufficient to control the bleeding [1]. Non-compressible hemostasis can significantly increase the chance of survival for injured people [2]. Currently, the United States Food and Drug Administration has confirmed the specific coagulation agents for different applications of which topical hemostatic agents play an important role in hemostasis [3]. Hemostasis after needle extraction at the end of each hemodialysis (HD) session in the dialysis ward is very important for patients, doctors, and nurses. The prolongation of bleeding time significantly affects the quality of life of patients and also increases the amount of work and time spent by the nursing staff [4]. The HD patients have coagulation problems, especially after HD, due to anemia, uremia, platelet activity impairment, and the use of heparin for HD. Most of these patients are also treated with anticoagulants and antiplatelets such as aspirin, heparin, and warfarin. Generally, strong direct pressure with conventional gauze dressing on the vascular access site of HD patients can stop bleeding. However, in some cases, bleeding may last for several minutes. Pressure for a long time can lead to clot formation and obstruction at the site of vascular access [5]. Oxidized regenerated cellulose is one of the hemostatic substances that accelerate the coagulation cascade and clot formation. Some research has been done on the effect of oxidized regenerated cellulose on other types of hemorrhages, but its efficacy in vascular access hemostasis has not been studied in HD patients. Due to some restrictions in the use of hemostatic materials in some types of injuries, new forms of this material have been designed [6]. HemoFoam® is a soft, flexible, non-allergic, biocompatible sponge with no genotoxicity, cell toxicity, skin irritation, sensitivity, and systemic toxicity (acute and subchronic toxicity). It can absorb blood up to 8 times of its weight (in about 2 min) [6]. Considering the mentioned information, the increasing need for better control of bleeding, and the need for improving the quality of life of HD patients, this study investigated the efficacy of HemoFoam® as a new form of hemostatic foam in hemostasis of dialysis access puncture wound bleeding in HD patients.

## Materials and Methods

### Study Design

This single-center, before-after, and the one-group clinical trial study was carried out in Shahid Rahnemoon Hospital in Yazd, Iran, 2017. Sixty patients undergoing HD via arteriovenous fistula (AVF) were enrolled in the study after passing the eligibility criteria.Considering a similar study [7], the sample size was set at 50, considering type I error of 0.05 and type II error of 20%, which was increased to 60 to enhance the validity of the study results. The trial was registered at the Thai Clinical Registry (TCTR) (http://www.clinicaltrials.in.th) with the TCTR ID: TCTR20180625001. This research was approved by the Ethics Committee of Shahid Sadoughi University of Medical Sciences (ethical code: IR.SSU.REC.1397.046).

### Inclusion And Exclusion Criteria

The inclusion criteria were all patients aged 18 to 70 years, HD via AVF three times a week, Glasgow Coma Scale of 15/15, and at least a six-month history of dialysis. The exclusion criteria were unwillingness to participate in the study, a vascular or bleeding disorder (coagulopathy), recent AVF angioplasty, patients treated with oral anticoagulants or antiplatelets, systemic infection symptoms, the absence of patients in the next HD sessions, and patient’s death during the study.

### Procedure

This study was performed in two separate sessions; only HemoFoam® was used during the first session, and only conventional gauze dressing in the second session in the hemostasis of the arterial or venous access site. Two nurses were invited to participate in the research. The purpose and protocol of the study were fully explained to them. The nurses performed the pressure (they were trained about the standardized manual pressure). In the first session, a questionnaire was used to record patient’s demographic data, blood pressure, history of diabetes, history of hypertension, the cause of end-stage renal disease, and bleeding time in HD patients who were enrolled in the study. The time required for hemostasis was also measured by a stopwatch (Casio HS-80TW-1DF Stopwatch® Casio, Japan). At the end of the HD session, the blood pressure of the patient was measured and recorded one-time by a standard procedure before the needles extraction (Diplomat, Sphygmomanometer 1002®, Riester, Germany). Sharp needles were used in all cases. Then, 2.8cc of blood was drawn from the venous access site and sent to the laboratory for platelet count, hematocrit, and coagulation tests (INR, PTT). The amount of heparin consumed during dialysis, isotonic saline solution volume for transfusion and blood products injected during dialysis were recorded. After HD was completed at the first session, as the needle was removed from the arterial access site, HemoFoam® (HemoFoam®, manufactured by Chitotech Co., Iran, diameter=22 mm, thickness=10 mm, weight=0.6 g) was placed at the access site. Slight manual pressure was applied (with two fingers) that was negligible. After two minutes, by lifting a corner of HemoFoam®, the needle removal site was examined for hemostasis or continued bleeding. The time for complete hemostasis (no leakage from the needle extraction site) was recorded, and 10 cm of adhesive tape was used to keep HemoFoam® in place. If hemostasis was not achieved after two minutes, manual pressure was applied again with two fingers, and this cycle continued every two minutes until hemostasis was achieved, as described above. After hemostasis achievement (secure hemostasis for at least three minutes) at the place where the needle was removed and recording the hemostasis time, all of the steps mentioned above were also performed for the second needle extraction site (venous site) and the time for homeostasis was recorded. At the next session (48 h later), before the start of HD, the patient was asked about any bleeding from the vascular access site or any use of anticoagulant, and the site was checked for hematoma. As the needles were removed (needles were removed after each HD session), a sterile gauze (2.5cm×2.5cm) and gentle manual pressure with two fingers were applied in the place of vascular access site as usual, and the other stages were similar to the HemoFoam® group. In the third session (48 h later), the site was examined for hematoma and recurrent bleeding.

### Statistical Analysis

All the required data were imported to SPSS22 (IBM SPSS Statistics for Windows, Armonk, NY: IBM Corp, United States) and analyzed by statistical tests including Chi-square and paired T-test. Paired T-test (or the equivalent nonparametric test, if applicable) was used to analyze the quantitative variables while Chi-square test was used to analyze the qualitative variables. The data were expressed as the mean±SD deviation (SD) and frequency (percentage). The significant statistical level was set at P=0.05.

### Ethical Considerations

The purpose of the study was explained to the patients, and the patient’s informed written consent was obtained. This research was approved by the Ethics Committee of Shahid Sadoughi University of Medical Sciences (in accordance with the Helsinki declaration). We did not perform any additional invasive procedures. All patient information remained confidential.

## Results

The results of the study showed that the mean age of the patients was 55.20±14.25 years. Thirty-five (58.33%) were male, and 25 (41.66%) were female (Table-1). Coagulation tests, hematocrit, platelet count, fistula location, history of diabetes, serum volume, and injected blood products did not change in different stages of the study. None of the patients needed blood transfusion, and none of them died during this study. Also, the mean duration of fistula function was 2.81±1.65 years (Table-2). The mean systolic blood pressure was 125.52±16.65 mmHg in the conventional gauze dressing group and 125.82±16.55 mmHg in the HemoFoam**®** group, which was not statistically significant (P=0.93, Table-3). The mean diastolic blood pressure was 79.68±10.13 mmHg in the conventional gauze dressing group and 78.88±9.85 mmHg in the HemoFoam**®** group, which was not statistically significant (P=0.38, Table-3). The mean time of hemostasis in the conventional gauze dressing group was 10.54±6.65 min at venous access site and 12.74±9.24 min at the place of the arterial access site. The mean hemostasis time was 2.86±1.87 min in the HemoFoam® group at the venous access site, and 3.15±1.97 min at the arterial access site, which decreased significantly in arterial and venous access sites compared to the conventional gauze dressing group (P<0.001, Table-4). In our study, hemostasis was achieved in 80% of cases in the first two min in the HemoFoam**®** group at the venous access site and in 76.6% of cases at the arterial access site. Hemostasis was achieved in 91.6% of cases after 4 min at the venous access site and in 90% of cases at the arterial access site. These differences were statistically significant compared to the gauze group. In the HemoFoam® group, there was no recurrent bleeding and hematoma formation after discharge. In the conventional gauze group, two patients (3.3%) had recurrent bleeding, and one (1.6%) patient had hematoma. HemoFoam® was also easily removed after discharge by all patients.

## Discussion

Hemostasis of dialysis access puncture wound bleeding after needle extraction is very important for HD patients. The common method of hemostasis of vascular access site of HD patients is using simple conventional gauze dressing and manual pressure [6-8]. Oxidized regenerated cellulose is one of the hemostatic agents that accelerate the coagulation cascade and clot formation. The HemoFoam® is mainly composed of oxidized regenerated cellulose, which can lead to blood absorption, increased coagulation factors in vascular access site, and platelet aggregation in the lesion, and then accelerates the formation of platelet plaques by creating a matrix for adhesion of the platelets [6]. HemoFoam® particles have a positive charge that absorbs the negative charge of the platelet and other blood cells and accelerates the formation of the clot [6]. The results of this study showed that the hemostasis achievement time was significantly lower with HemoFoam® compared to the conventional gauze dressing. Kordestani *et al.* carried out a study on 124 patients undergoing coronary artery angiography. They found that ChitoHem® powder (composed mainly of oxidized regenerated cellulose) reduced the hemostasis time at the vascular access site of coronary artery angiography more effectively than manual pressure [6]. The hemostasis time was 4.6 min in the ChitoHem® group and 12.4 min in the control group, which is consistent with our study findings. The reason for this difference can be due to high arterial pressure in the femoral artery and an increase in femoral artery bleeding over the AVF. HemoFoam® used in our study is mainly made of oxidized regenerated cellulose. In our study, there was a 9.59-min reduction in hemostasis time at arterial access site using HemoFoam® compared to conventional gauze. However, in Kordestani *et al.* study [6], there was a 7.8-min reduction in hemostasis time using ChitoHem® powder at the site of angiographic vascular access compared to manual pressure. Also, Misgav *et al*. evaluated the effect of hemostatic Chitosan-based pads compared to the gauze pads in local hemostasis. Fifteen patients who had prolonged hemostasis time after needle extraction were enrolled. At the end of each dialysis, either gauze or Chitosan pad was applied on vascular access sites. The mean hemostasis time was significantly lower in the Chitosan-based pads group than in the normal gas group. The authors concluded that Chitosan-based pads could be effective in bleeding control in patients with coagulopathy. In the study by Misgav *et al*., the hemostasis time was 3 min at the arterial access site in the Chitosan pad group compared to 18.5 min in the control group while this time was 2.8 min at the venous access site in the Chitosan group compared to 13.2 min in the control group [8]. This difference can be due to two reasons. Misgav *et al.* conducted a study on high-risk patients (patients with prolonged hemostasis time), but our study was carried out on all HD patients. This difference can also indicate a better efficacy of HemoFoam® than the Chitosan-based pads.The study by Bachtell *et al.,* aimed to evaluate the time of hemostasis after using Chitosan-based bandage (HemCon®), was carried out on 50 patients. The hemostasis time was measured in the second and fourth min. It was determined that hemostasis was achieved after 2 min in 30% of patients with the Chitosan-based bandage and in 38% of patients with conventionaldressing and after 4 min in 86% of patients treated with Chitosan-based bandage and 72% of patients with conventional dressing. They concluded that Chitosan-based bandage was an effective and safe hemostatic dressing that reduces bleeding from the puncture wound after HD [7]. Moreover, Boulanger *et al.* carried out a study during three weeks with the aim of evaluate the safety and efficacy of IRIS® bandage (Nephrokit®) in comparison with the common manual pressure in dialysis access puncture wound bleeding. Common manual pressure was used during the first week, IRIS® bandage during the second week, and common manual pressure during the third week. The difference between conventional manual pressure and IRIS® bandage was statistically significant [4]. In our study, hemostasis was achieved in 80% of cases at the venous access site in the first two min in the HemoFoam**®** group and 76.6% of cases at the arterial access site. The difference between HemoFoam**®** and conventional gauze dressing was significant in venous and arterial access sites in the second and fourth minutes. The results of this study are consistent with the studies of other researchers [4, 6, 7]. In this study, the use of HemoFoam® did not cause any local skin reaction. In Bachtell *et al*. study, there was no complication at vascular access site in Chitosan-based bandage (HemCon®) group. There was no local skin reaction with Chitosan pad in the Misgav *et al*. study. In Kordestani *et al.* study, there were no complications regarding the use of ChitoHem® at the site of angiography access [6]. Also, the study by Unalp *et al*. showed that the use of poly-n-acetyl glucosamine patch reduces the incidence of thrombosis in the vascular access site [5]. The price of just one HemoFoam® is about 2.5 times higher compared to conventional gauze. However, HemoFoam® is more effective in reducing the time of homeostasis in HD patients. In some cases, with the use of conventional gauze dressing, we have to use several gauzes to achieve hemostasis. Finally, the decrease in the duration of manual pressure can be one of the important factors in reducing complications (including thrombosis). In the present study, HemoFoam® also reduced the duration of hemostasis time significantly, which can probably lead to a reduction in the possible side effects of prolonged manual pressure. The strong point of our study was the number of patients. Each patient also had self-control, the importance of which was to compare HemoFoam® and common gauze in the same conditions. The authors did not consider the carry-over effect from the first to the second session. However, regarding the topical use of HemoFoam®, the carry-over effect from the first to the second session is difficult to imagine, but it cannot be excluded. One of the limitations of this study was that the researcher had to remove the pressure every two minutes to evaluate the hemostasis time, which would interfere with the process of thrombosis. However, in this study, we tried to do this by quickly lifting up part of the conventional gauze dressing or HemoFoam®. In the HD Ward of Shahid Rahnemoon Hospital, where this study was conducted, most of the patients had AVF, and only a limited number of the patients had the graft. Considering the risk of hemorrhage and prolonged hemostasis time at vascular access site in patients with graft [9], further studies on these patients is recommended. It is also recommended to evaluate the effect of HemoFoam**®** on the incidence of thrombosis in HD patients and long-term skin reactions. Regarding the carry-over effect, in future studies, it is suggested that the control group be performed first.

## Conclusion

Given the findings of this study, HemoFoam® significantly reduces hemostasis time. It is also effective and safe in reducing bleeding at the vascular access site of HD patients. Therefore, its use is recommended in HD wards for achieving hemostasis at the vascular access site of HD patients.

## Acknowledgment

The authors would like to thank all the patients and their families who participated in the study. We would also like to acknowledge Baqiyatallah University of Medical Sciences (Tehran, Iran) and Shahid Rahnemoon Hospital (Yazd, Iran). This manuscript is distilled from the thesis of Rouhollah Zarepur instead of the military service in accordance to Bonyad Melli Nokhbegan (BMN) regulations. We want to thank Dr. Ehsan Zarepur for his help in design, analyze the data, and writing this article.

## Conflict of Interest

None declared.

**Table 1:** Baseline Demographic and Clinical Characteristics of HD Patients Treated with HemoFoam® or Conventional Gauze Dressing

**Variables**	**Subgroup**	**n (%) or Mean ± SD**
**Age (years)**	-	55.20±14.25
**Sex**	Male	35 (58.33)
	Female	25 (41.66)
**Diabetes**	Yes	42 (70.00)
	No	18 (30.00)
**History of hypertension (mmHg)**	Yes	34 (56.66)
	No	26 (43.33)
AVF location*	Wrist	32 (53.33)
	Elbow	28 (46.66)

*AVF: Arteriovenous fistula

**Table 2:** Characteristics of HD Patients Treated with HemoFoam® or Conventional Gauze Dressing

**Variables**	**Mean ± SD** **(n=60)**
**AVF vintage** ^*^	2.81 ± 1.65
**HD vintage** ^**^	8.5±3.10
**Prothrombin time PT, min**	12.90 ± 0.99
**Partial thromboplastin time (PTT), min**	34.01 ± 3.44
**International normalized ratio (INR), IU**	1.27 ± 0.17
**Platelet count, per cubic millimeter**	195000 ± 31113.80
**Haematocrit, %**	31.79 ±3.20

*AVF: Arteriovenous fistula; **HD: Hemodialysis

**Table 3:** Comparison of The Mean Blood Pressure and Heparin Levels in HD Patients Treated with HemoFoam® or Conventional Gauze Dressing

**Variables**	**Conventional gauze dressing** **(Mean ± SD)**	**HemoFoam®** **(Mean ± SD)**	**P-value**
**Systolic blood pressure (mm Hg)**	125.52 ± 16.65	125.82 ± 16.55	0.93
**Diastolic blood pressure (mm Hg)**	79.68 ± 10.13	78.88 ±9.85	0.38
**Heparin (IU)**	5500 ± 588.25	5525.25 ± 508.43	0.33

**Table 4:** Comparison of The Mean Hemostasis Time in HD Patients Treated with HemoFoam® or Conventional Gauze Dressing

**Variables**	**Time to hemostasis (min)** **Mean ± SD**	**P-value**
**HemoFoam® (arterial)**	3.15 ± 1.97	<0.001
**Conventional gauze dressing (arterial)**	12.74 ± 9.24	
**HemoFoam® (venous)**	2.86 ± 1.87	<0.001
**Conventional gauze dressing (venous)**	10.54 ± 6.65	
